# Depression, a Hidden Mental Health Disparity in an Asian Indian Immigrant Community

**DOI:** 10.3390/ijerph13010027

**Published:** 2015-12-23

**Authors:** Lisa R. Roberts, Semran K. Mann, Susanne B. Montgomery

**Affiliations:** 1 School of Nursing, Loma Linda University, 11262 Campus Street West Hall #1327, Loma Linda, CA 92350, USA; 2 School of Behavioral Health, Division of Interdisciplinary Studies, Loma Linda University, 11065 Campus Street, Loma Linda, CA 92350, USA; skmann@llu.edu (S.K.M.); smontgomery@llu.edu (S.B.M.)

**Keywords:** Asian-Indian, immigrants, gender, mental health, acculturation, Punjabi Sikhs

## Abstract

Cultural influences are deeply rooted, and continue to affect the lives of Asian-Indian (AI) immigrants living in Western culture. Emerging literature suggests the powerful nature of traditions and culture on the lives, mental and physical health of AI immigrants, particularly women. The purpose of this study was to explore depression among AI women in Central California (CC). This mixed-methods research was conducted in collaboration with the CC Punjabi community and the support of local religious leaders. All interviews were conducted in Punjabi and English. Whenever possible we utilized validated scales aligned with emerging themes from the qualitative data, which also provided contextualization to survey responses. In all we conducted 11 key informant interviews, four focus groups (*n* = 47) and a rigorously developed anonymous survey (*n* = 350). Social dynamics and traditional expectations including gendered roles significantly affected mental health among women participants. Subgroups along the lines of language choice (Punjabi *vs.* English) experience and report depression differently in part due to the highly stigmatized nature of mental health issues in this model minority community. The findings of this study highlight the importance of utilizing mixed methods to access hard to reach populations regarding sensitive topics such as mental health.

## 1. Introduction

Asian-Indians (AIs) constitute one of the fastest growing minority groups in the U.S. [1]. Currently there are over three million AIs in the U.S. [2], with many AI immigrants settling in California [3,4], including Punjabis, Gujaratis, Bengalis, and Tamils [5,6]. Although this rapid growth is well documented, literature is lacking on AI mental health needs and preferences for care in the U.S. [7].

Immigration is fraught with challenging issues while settling into a new country and culture, including limited language proficiency, difficulty navigating a new educational system, finding employment, accessing the health care system, adjusting to minority status, facing discrimination and for AI immigrants—expectations of living up to the model minority myth, putting them at risk for mental health consequences [8,9,10]. An understanding of the mental health of AI immigrants must take into account health beliefs, economic and physical environment, social support of family and community, as well as culture and gender [11], as well as diversity between subgroups of AI immigrants [8,12]. For instance while AI subgroups share several cultural tenants such as son preference and patriarchy, Punjabi Sikh AI have very different religious guiding principles with respect to equality across the community, than those who are Hindu where the caste system remains pervasive [13].

In addition to the typical acculturation issues, AI women in particular may face unique challenges when immigrating to the U.S. AI immigrant women face multiple facets of inequality and discrimination within and outside of the AI community [14]. Some AI immigrant women are less educated than the model minority stereotype would suggest, having come to the U.S. as wives of AI immigrant men, and may then be confined to the home upon arrival [15]. Even AI women seemingly more connected to U.S. culture by getting advanced education often experience similar challenges. A recent study [16] noted that Indian nursing students might erroneously be considered independent and autonomous based on their skill level and immigration status. However, in actuality, these women were found to be under the care and containment of their distant families in India, who had orchestrated their migration for the improved socio-economic status of the family or collective interests.

Second generation AI immigrant women (of foreign born parent/s) [17] have been found to be particularly prone to cultural value conflict and high maternal control, contributing to depression. Women are expected to balance their immigration and its intrinsic “freedom” by maintaining gendered patriarchal values, demonstrated by their dedication to their responsibilities of home and husband, thus safe-guarding family honor. Fear of social penalty, being disavowed by their families, or violence if perceived to be behaving inappropriately is distressing to AI immigrant women [16].

Though it may seem that AI women in the U.S. are somewhat “freed” of the pressure traditional women in India face, they are keenly aware of high expectations of their new families in the U.S. as well as from back home in India. Indeed, families in India often fear that the women will conduct themselves dishonorably due to the perception that with emigration they will inherently have increased mobility and engagement with Western society [16] and begin to adopt some of what are considered less desirable values and behaviors. However, in reality, AI immigrant women often face a controlling spouse and in-laws instead of newfound freedom. Upon arrival they may be forced to find a job, turn over their earnings, or suffer physical abuse, social isolation, or desertion, causing mental and emotional distress [18].

These immigration related elements can be seen as risk factors for mental health issues, yet relatively low rates (approximately 3%) of depression have been identified among a general pool of new U.S. immigrants, though women were significantly more likely to indicate depression [10]. Depression has also been noted as a major public health issue specifically among AI immigrant women in the U.S. [19]. Suggested causes in the very limited literature on this issue provided by a Canadian study include family and relationships, as well as culture and migration, and socioeconomic issues [18]. With few studies exploring depression among AI immigrants [15,20] we have a limited understanding of risk factors, predictors, and possible solutions of depression in this group of immigrants.

AI women may be reluctant to report mental health issues or may not recognize symptoms as related to an underlying mental health issues [21]. Psychological symptoms are stigmatized as conveying weakness and personal deficiencies that are shameful and bring dishonor to the family. Therefore, rather than seeking mental health care, individuals may present with physical symptoms if any care is sought. Somatization is common among AIs experiencing psychological distress [15]. However, self-blame and social norms engendering stoic forbearance of psychosocial distress often prevent AI individuals from seeking care. Unfortunately, AIs with psychological distress fear stigmatization to the extent of becoming isolated even from their own community where social support might otherwise be sought [1,18].

Punjabi AI immigrants are primarily Sikhs, coming from Punjab, a largely rural agricultural region and as such the majority of California-settled Punjabi AI have a farming background [22]. Sikhism, regardless of occupation or economic status, is an integral part of the Punjabi community. Religious beliefs and social expectations, including those governing family and community relationships, are factors that may influence AI immigrants daily living, interpretation of new experiences, and health outcomes [23]. As reported previously [21], we found that while overall reporting of mental health issues was low, depression was significant among women in the Punjabi community. As this was an exploratory study we had not preselected any subgroups, rather explored patterns within the Punjabi community as a whole. The purpose of this study was to further explore depression among Punjabi AI women in Central California. We hypothesized that social expectations regarding gendered roles and acculturation would be significant to mental health.

## 2. Experimental Section

### 2.1. Methods

#### 2.1.1. Participants

A total of 58 persons participated in the qualitative phase. Key informant interviews (*n* = 11) with four men and seven women of different ages were conducted; included were community stakeholders, such as mental health and other health care providers, religious leaders, and also general community members. Four focus groups consisted of (a) AI married men (*n* = 18), (b) married AI women of reproductive age (*n* = 14), (c) four AI women involved in a Sikh women’s advocacy group, and (d) AI mother-in-law’s (*n* = 11). Focus group participants varied in socio-economic status, educational background, employment, and age.

**Table 1:** All survey participants (*N* = 350).

Characteristic	*n* (%)
Marital status	
Single	74 (21.1)
Married	256 (73.1)
Widowed	7 (2.0)
Separated/divorced	6 (1.7)
Education	
No high-school diploma	18 (5.1)
High-school diploma	61 (17.4)
Some college	88 (25.1)
≥Bachelor’s degree	172 (49.1)
Employment **^a^**	
Employed	198 (56.6)
Unemployed	150 (42.9)
Birth place	
U.S.	46 (13.1)
India	293 (83.7)
Other **^b^**	9 (2.6)
Living in a joint family **^c^**	
Yes	187 (53.4)
No	123 (35.1)
	*M* (SD)
Age	41.85 (15.38)
Years Married	32.98 (25.20)
Age Married	24.04 (3.82)
Years in U.S.	18.62 (10.78)
Assimilation	0.72 (1.40)
Separation	2.77 (2.47)
Integration	4.32 (2.46)
Marginalization	0.14 (0.64)
Attitudes towards women	30.83 (6.98)
Domestic violence myths	3.53 (1.14)
Positive religious coping	5.36 (2.38)
Negative religious coping	8.43 (2.71)
Overall religiosity	1.67 (0.80)
Satisfaction with life	23.10 (8.87)
Anxiety	5.49 (5.27)
Depression	5.04 (5.32)

**^a^** Some participants who indicated they were unemployed farmed their own land or were otherwise self-employed; **^b^** Other countries of birth were included but all participants self-identified as Asian-Indian; **^c^** A joint family includes the parents of the husband living with the husband and wife.

Quantitative (*N* = 350): Participants included 217 (62%) women and 133 (38%) men, with a total of 268 (76.6%) completing the survey in English. The vast majority (73.1%) were married, and highly educated (74.2% having had some college or completed at least a bachelor’s degree). Most were born in India (83.7%), and of the mean age of participants was nearly 42 years old while the average number of years in the U.S. was under just under 19 years. See Table 1 for details of participant characteristics.

#### 2.1.2. Materials

Semi-structured outlines for key informant interviews were developed based on the literature and planning discussions with community stakeholders. Semi-structured outlines for focus group discussions were developed from the themes that emerged from the early key informant interviews. Surveys included descriptive demographics deemed important based on qualitative data, including place of birth, and questions pertaining to: (a) ideal family composition, (b) family planning experiences, (c) joint family living arrangements (with her husband and his parents), and (d) social norms regarding ideal family structure. Additional items for women only pertained to obstetric history to validate the qualitative findings of gendered role expectations. Selection and inclusion of validated scales were guided by the qualitative results.

### 2.2. Procedures

Institutional Research Board (IRB) approval from Loma Linda University as well as from community stakeholders was received before the study commenced. Due to the lack of literature regarding depression among AI immigrants, and even less for the Punjabi sub-group, we employed a mixed-methods design. Punjabi AI adults in the central valley of California were recruited to participate in qualitative aspects of the study initially by convenience sample of community stakeholders at a Punjabi AI health fair. Snowball technique was then used to recruit additional key informants, for a total of 11 semi-structured key informant interviews and four focus groups that were conducted throughout the study. Informed consent was obtained prior to each interview. Key informant interviews were conducted using semi-structured outlines based on literature regarding AI sociocultural determinants of health, to ensure consistent exploration of issues. Key informants included Punjabi AI men and women of varying age and socioeconomic status. Key informant interviews were conducted by the second author, an AI immigrant women, for participants who were most comfortable speaking in Punjabi. The first author interviewed the male participants, who all spoke English. This arrangement was also respectful of social norms by avoiding putting the second author in an inappropriate sociocultural interaction as it would not be typical for a young, single Punjabi woman to ask personal questions of older and/or married men. On average interviews were 45 min long.

Four focus groups (*n* = 47) were conducted using a semi-structured outline. The main purpose of these focus group discussions were to further explore and validate the emerging themes in a social context allowing open discourse and contextualization. Focus groups were conducted in English with the help of a Punjabi speaking co-moderator for immediate translation of key words or concepts as needed. Topics included traditional roles and social expectations, reproductive ideals and choices, experiences influenced by gender, and effects on health. Age, family role, and participation in a special interest group defined groups of women for three of the focus groups. Men had a separate focus group and included married men with various occupations.

Triangulation of some of the initial key informant interviews were used to obtain a broad perspective of views and convergent validation [24] which guided the design of an aligned survey. These and later conducted qualitative interviews were also used to help shed contextual light on emerging quantitative issues. Field notes were also taken for contextualization and reflection throughout the study.

Participants for the quantitative portion (*n* = 350) of the study were recruited during community events at *Gurdwaras* (Sikh churches) to participate in the anonymous survey, which was made available in either English or Punjabi based on the qualitative results that suggested that many of our respondents were more comfortable speaking and reading in Punjabi. Independent forward and backward translation of the survey was completed to ensure cultural and functional equivalence rather than relying only on literal translation [25].

### 2.3. Measures

#### 2.3.1. Satisfaction with Life Scale (SWLS)

The SWLS is a 5-item scale, rated (1) strongly disagree, to (7) strongly agree, designed to assess overall life satisfaction as a whole [26] with high reliability and validity [27]. The items are summed, with a score of 30 to 35 is very high, indicating very highly satisfied with life; 25–29 is a high score, indicating high satisfaction; 20–24 is an average score; 15–19 indicates slightly below average life satisfaction; 10–14 indicates dissatisfaction; and a score of 5–9 indicates extremely dissatisfied with life [28]. This scale was chosen for its simplicity and previous use among Indian women [29]. Previous studies have indicated reliability with Cronbach’s alphas of .80 to .96 [30]. In the current study it was 0.96.

#### 2.3.2. Acculturation, Habits, and Interests Multicultural Scale for Adolescents (AHIMSA)

AHIMSA is composed of eight items with response options for all items: (a) The United States, (b) The country my family is from, (c) Both, (d) Neither. The scale generates four scores ranging from 0 to 8 for Assimilation (“United States” responses), Separation (“The country my family is from” responses), Integration (“Both” responses), and Marginalization (“Neither” responses). The scores are not summed, rather the researcher must select the highest score as the participant’s orientation. Previously the AHIMSA scale showed good reliability with a Cronbach’s alpha of 0.84 [31]. Although created for adolescents, this scale was chosen by community stakeholders for its brevity and as most intuitive in their cultural context when compared to other acculturation scales. The current study using AHIMSA among AI adults has an acceptable Cronbach’s alpha of 0.70.

#### 2.3.3. Short Attitudes toward Women Scale (AWS)

The 15-item version of the AWS, developed by from the 25-item version [32], and later further validated [33]. Response options are (a) agree strongly, (b) agree mildly, (c) disagree mildly, (d) disagree strongly. The scale was subsequently further shortened to 12 items and validated among Turkish students, with a reported Cronbach’s alpha of 0.83 [34]. Seven items are reverse coded and then all items are summed, for a possible score of 0 to 36. A high score indicates a pro-feminist, egalitarian attitude, whereas a low score indicates a traditional, conservative attitude. Cronbach’s alpha for our current study was 0.88. This scale was chosen to shed light on gender discrimination.

#### 2.3.4. Domestic Violence Myth Acceptance Scale (DVMAS)

DVMAS is an 18-item scale with a 7-point Likert response option ranging from (1) strongly disagree to (7) strongly agree for the first 17 items and (1) not at all; to (7) entirely; for the last item. All 18 items are summed and then divided by 18 for a mean score; resulting in scores ranging from 1–7 [35]. Higher scores indicate blaming the victim; exonerating the perpetrator; and minimizing the violence; which follow gendered norms. This scale has performed well in divergent populations; including India; Japan; and Argentina (Jay Peters; personal communication; 21 August 2013). Peters [35] reported reliability of 0.81 to 0.88; and similarly in the current study our Cronbach’s alpha was 0.82. We included this scale to explore beliefs regarding domestic violence in the Punjabi AI community.

#### 2.3.5. Short Form of the Brief RCOPE

This is a 7-item version of the religious coping measure, using six items rated on a Likert-type scale ranging from (0) not at all, to (3) a great deal for both positive and negative religious coping. The seventh item measures the extent that religion is used to understand or deal with stressful situations with response options (0) not involved at all, to (3) very involved [36]. The scale is built on a monotheistic paradigm, which pertains to Sikhism as well. Similar to previous studies among non-Western cultures with religions other than Christianity reporting Cronbach’s alphas of 0.60 to 0.75 [37], the current study had a reliability of 0.71.

#### 2.3.6. Patient Health Questionnaire 9 (PHQ-9)

The PHQ-9 is a depression screener containing nine items with response options (0) not at all, (1) several days, (2) more than half the days, and (3) nearly every day. Scores are summed for a possible score of 0 to 27 and cut points of 5, 10, and 15 represent mild, moderate, and severe levels of depression [38,39]. There are numerous translations available, including Punjabi. This brief questionnaire is reported to be useful in both the clinical and research setting, with Cronbach’s alphas of 0.86 to 0.89, indicating good reliability [38,39]. Cronbach’s alpha was 0.82 in the current study.

#### 2.3.7. General Anxiety Disorder-7 (GAD-7)

The GAD-7 is a seven-item anxiety measure utilizing a 4-item Likert-scale response option ranging from (0) not at all, (1) several days, (2) more than half the days, and (3) nearly every day. The items are summed for a possible score of 0 to 21, with 5, 10, and 15 represent mild, moderate, and severe levels of anxiety. This is a useful clinical and research tool with good reliability as indicated by reported Cronbach’s alpha of 0.92 [40]. In the current study, the Cronbach’s alpha was 0.92 and as the GAD-7 is already available in English and Punjabi, it did not require translation. This scale, as well as PHQ-9, were chosen to elucidate qualitative responses alluding to mental health issues.

### 2.4. Analysis

All key informant interviews and focus groups were audio recorded and transcribed verbatim. Punjabi transcripts were translated into English. Open coding followed by axial coding and thematic analysis using Grounded Theory principles was completed on all transcriptions as reported elsewhere [41,42]. For the purposes of this study, selective coding [43] for the purpose of contextualizing the quantitative results was utilized to further explore the themes related to depression. These themes validated by quotes were then discussed until consensus was reached by the three authors.

Quantitative data analysis was accomplished using SPSS. Analysis included descriptive statistics, factor analysis, significance tests between sets of variables of interests, and multiple linear regression equations. Data was collected from 350 surveys.

## 3. Results

### 3.1. Preliminary Analysis

On average, women were significantly younger than men at the time of marriage, had been married a shorter amount of time, were less likely to be employed, and more likely to have been born in India. As noted in our previous work [21], the mental health variables were significantly different between genders; on average, women indicating elevated levels of anxiety and depression while men indicated levels within the normal range. Therefore, in the current study we first explored participants by survey language preference within gender groups. Numerous significant differences were found for participants choosing to complete the survey in Punjabi *vs.* English in both gender groups. Further descriptive details are given in Table 2. Depression was highest among women who chose to complete the survey in Punjabi, with a mean of 7.73, indicating mild to moderate levels of depression. All other subgroups had means within the normal range (<5).

### 3.2. Bivariate Analysis

Since depression was more of an issue for women, we then explored correlates of depression among women who completed the survey in English and in Punjabi. Among women who completed the survey in English, ideal family size, negative religious coping, and anxiety were significantly correlated with depression. Among women who completed the survey in Punjabi, age at marriage, age at first pregnancy, domestic violence myth acceptance, and anxiety were significant correlates of depression (see Table 3).

### 3.3. Multiple Regression

All significant correlates of depression in women were explored using multivariate analysis in a stepwise fashion. Among women who completed the survey in English, the first step included ideal family size and negative religious coping, which explained 12.7% of the variance and both variables remained significant. When anxiety was added in the second step, 64.7% of the variance was explained; ideal family size was no longer significant, negative religious coping although still significant had decreased impact with anxiety overpowering all.

Among women who completed the survey in Punjabi the first step, including age married, age at first pregnancy, and domestic violence myth acceptance, explained 51.9% of the variance and age at first pregnancy remained significant. Anxiety was added in the second step, resulting in an increase to 56.8% of the variance explained, however, age at first pregnancy was no longer significant—only anxiety was a significant predictor (see Table 4).

**Table 2:** Comparing by survey language within gender groups.

Characteristic	Women		Men	
	Punjabi (*n* = 52)	English (*n* = 165)	Punjabi (*n* = 30)	English (*n* = 103)
	*n* (%)		*n* (%)	
Marital status				
Single	5 (9.6)	22 (13.3)	3 (10.0)	44 (42.7)
Married	41 (78.8)	133 (80.6)	26 (86.7)	56 (54.4) *
Widowed	3 (5.8)	3 (1.8)	1 (3.3)	0
Separated/divorced	0	4 (2.4)	0	2 (1.9)
Education				
No high-school diploma	5 (9.6)	8 (4.8)	2 (6.7)	3 (2.9)
High-school diploma	15 (28.8)	15 (9.1)	13 (43.3)	18 (17.5)
Some college	20 (38.5)	32 (19.4)	9 (30.0)	27 (26.2)
≥ Bachelor’s degree	11 (21.2)	104 (63.0) **	3 (10.0)	54 (52.4) **
Employment **^a^**				
Employed	22 (42.3)	88 (53.3)	17 (56.7)	32 (31.1)
Unemployed	29 (55.8)	77 (46.7)	12 (40)	71 (86.9)
Birth place				
U.S.	3 (5.8)	18 (10.9)	1 (3.3)	24 (23.3) *
India	48 (92.3)	140 (84.8)	29 (96.7)	76 (73.8)
Other **^b^**	0	6 (3.6)	0	3 (2.9)
Living in a joint family **^c^**				
Yes	41 (78.8)	75 (45.5) ***	26 (86.7)	45 (43.7) ***
No	7 (13.5)	70 (42.4)	3 (10.0)	43 (41.7)
	*M* (SD)		*M* (SD)	
Age	45.67 (13.42)	41.01 (12.82) *	53.90 (16.22)	37.78 (17.63) **
Years Married	22.96 (13.09)	19.11 (11.86)	31.77 (16.33)	20.22 (14.85) *
Age Married	23.29 (4.31)	24.00 (17.39)	24.81 (4.36)	26.16 (5.32)
Years in U.S.	15.02 (10.47)	19.61 (10.42) *	14.72 (10.72)	19.97 (11.02) *
Assimilation	0.38 (1.11)	0.69 (1.27)	0.24 (0.44)	1.08 (1.78) **
Separation	3.60 (2.42)	2.56 (2.29) **	4.55 (2.94)	2.19 (2.35) **
Integration	3.48 (2.34)	4.64 (2.34) **	2.76 (2.53)	4.66 (2.44) **
Marginalization	0.40 (1.11)	0.09 (0.60)	0.21 (0.56)	0.08 (0.31)
Attitudes towards women	27.38 (5.70)	32.19 (6.33) ***	25.60 (3.37)	31.67 (7.78) ***
Domestic violence myths	3.87 (0.90)	3.34 (1.14) *	4.28 (1.02)	3.49 (1.19) *
Positive religious coping	6.19 (2.59)	4.95 (2.19) **	4.70 (1.92)	5.67 (2.49)
Negative religious coping	7.80 (2.56)	8.45 (2.89)	8.34 (2.71)	8.70 (2.52)
Overall religiosity	2.06 (0.97)	1.51 (.68) **	1.65 (0.94)	1.69 (0.78)
Satisfaction with life	15.88 (8.83)	26.23 (7.28) ***	16.64 (8.78)	23.92 (8.18) ***
Anxiety	7.19 (5.16)	6.00 (5.32)	4.36 (4.56)	4.37 (5.19)
Depression	7.73 (6.19)	4.89 (5.24) **	3.42 (3.96)	4.39 (4.94)

**^a^** Some participants who indicated they were unemployed farmed their own land or were otherwise self-employed; **^b^** Other countries of birth were included but all participants self-identified as Asian-Indian; **^c^** A joint family includes the parents of the husband living with the husband and wife; * = *p* < 0.05, ** = *p* < 0.01, *** = *p* < 0.001.

**Table 3:** Bivariate analyses among women (2-tailed).

Characteristics Correlated with Depression	English *n* = 126	Punjabi *n* = 45
Age	−0.139	−0.144
Employed	−0.123	−0.010
≥Bachelor’s degree	−0.150	0.294
U.S. Born	0.031	0.062
Years in the U.S.	−0.100	−0.177
Ever or currently married	0.129	−0.269
Age married	0.106	0.417 **
Years married	0.122	0.243
Living in joint family	0.023	−0.237
Ideal family size	0.200 *	0.001
Ideal number of boys	0.146	−0.047
Ideal number of girls	−0.075	0.139
Age at first pregnancy	−0.166	−0.324 *
Assimilation	0.017	−0.058
Separation	0.018	−0.039
Integration	−0.023	0.129
Marginalization	0.010	−0.164
Positive religious coping	−0.163	0.286
Negative religious coping	−0.298 **	−0.174
Overall religiosity	−0.058	0.126
Attitudes towards women	−0.039	−0.075
Domestic violence myths	−0.060	−0.333 *
Satisfaction with life	−0.143	0.031
Anxiety	0.770 ***	0.763 **

* = *p* < 0.05, ** = *p* < 0.01, *** = *p* < 0.001.

**Table 4:** Predictors of depression among AI immigrant women.

**Women Who Did the English Survey**	**Depression**			
Model 1 (*r^2^* = 0.127)	*B*	*SE B*	β	*95% CI*
Ideal family size	2.04	0.75	0.258 **	0.55–3.52
Negative religious coping	−0.52	0.18	−0.280 **	−0.86–−0.17
Model 2 (*r^2^* = 0.647)	*B*	*SE B*	β	*95% CI*
Ideal family size	0.49	0.51	0.061	−0.52–1.49
Negative religious coping	−0.28	0.12	−0.149 *	−0.51–−0.04
Anxiety	0.79	0.07	0.753 ***	0.65–0.92
**Women Who Did the Punjabi Survey**	**Depression**			
Model 1 (*r^2^* = 0.519)	*B*	*SE B*	β	*95% CI*
Age married	0.41	0.30	0.317	−0.20–1.02
Age at first pregnancy	−0.84	0.38	−0.505 *	−1.62–−0.06
Domestic violence myth acceptance	−0.86	1.33	−0.116	−3.57–1.86
Model 2 (*r^2^* = 0.568)	*B*	*SE B*	β	*95% CI*
Age married	0.05	0.24	0.041	−0.44–0.55
Age at first pregnancy	−0.43	0.31	−0.259	−1.06–0.20
Domestic violence myth acceptance	0.15	1.04	0.020	−1.98–2.28
Anxiety	0.82	0.18	0.664 ***	0.45–1.19

* = *p* < 0.05, ** = *p* < 0.01, *** = *p* < 0.001.

### 3.4. Qualitative Results

As noted above, our quantitative results clearly point to differences in the English *vs.* Punjabi survey selecting women’s experiences of depression. Among the women who took the survey in English the mean for depression was within normal limits (<5), however, when these women did experience depression it was associated with ideal family size, negative religious coping, and anxiety. Among women who completed the survey in Punjabi the mean for depression (7.73) was in the mild to moderate range (>5, <10) and age married, age at first pregnancy, and views around domestic violence were contributors to depression, which were completely overwritten by anxiety in a multivariate context. The qualitative results are less clearly delineated by language, as most women used a mixture of English and Punjabi during key informant interviews and focus group discussions, however, some women only spoke Punjabi. Women who were comfortable speaking English expressed a difference between themselves and those that only spoke Punjabi, who were generally older women (mother-in-laws) or younger wives who were recent immigrants. And vice-versa, women who only spoke Punjabi pointed out differences between themselves and AI women who spoke primarily English. Thus, we believe language serves as a proxy for acculturation. The qualitative results are summarized in three themes: (a) acculturative difference by language, (b) marriage and family conflicts, and (c) domestic abuse issues.

#### 3.4.1. Acculturative Difference by Language

Women who participated in English were more often second-generation AI immigrants than women who participated in Punjabi. Those who participated in English often discussed family conflict in terms of acculturative-intergenerational stress whereas women who participated in Punjabi who discussed family conflict did so in terms of adjusting to fit the expectations of her joint family.

Women who participated in English sometimes felt stifled in the joint family context, expressing a lack of freedom to be ones’ self, having to rely on having a progressive-minded husband to bridge the gap between her desires and the in-laws expectations. Some also voiced the intent to do things differently with their own children. This intent was voiced as an obligation and a hope, to reduce the acculturative stress that seemed to exacerbate the generation gaps at both ends of the continuum. As second-generation AI immigrants they were acutely aware of the expectations of the older generation (their parents and in-laws) as well as the younger generation (their own children). Women expressed tension noted in their own experiences of trying to balance protecting ones’ reputation/family/community according to AI gendered expectations with engaging in social relationships within the U.S. context.

Our Punjabi speaking participants expressed security within the AI community, they were able to engage in social obligations, religious activities, and function in their family roles, however, they were also isolated by language, rarely going outside the AI community without being accompanied by a bilingual AI immigrant—usually their husband. These women expressed the that their husbands could either help diffuse the pressure they felt to meet expectations of their in-laws and the AI community or if the husband was less involved, the women felt they had to work out these tensions for themselves. While expressing their devotion to family and home responsibilities, some also expressed a sense of loss (family left in India, educational/professional pursuits, freedom of movement), and a lack of decision-making power regarding anything outside of her household responsibilities.

#### 3.4.2. Marriage and Family Conflict

All women regardless of whether they were English or Punjabi speaking discussed issues regarding the expectation of an arranged marriage, appropriate age for marriage, pressure to have children soon after marriage, and childrearing responsibilities as part of an AI woman’s role regardless of immigration status. Son preference was expressed as a taboo subject but acknowledged as still present in the community.

Women expressed traditional gendered roles as a given expectation whether or not they also worked outside of the home. They acknowledged that this role often included living in a joint family (unless the husband’s family lived elsewhere) and being subservient to both husband and in-laws, while no longer actively caring for one’s own parents. Many women expressed a perceived lack of social support, and loneliness, often discussing how their mothers were different than their mother-in-laws, their frequent contact with natal kin by phone or internet (though sometimes restricted to less than what they desired), and that they were expected not to spend too much time with friends as that might “look bad”. Traditional gendered roles were discussed as important to preserving family honor.

Women who worked outside of the home discussed the expectations surrounding their employment. Most often they were given a job to do which supported a business within the extended family. Yet it was expressed that their contribution is not acknowledged or perceived to be appreciated, but rather an understanding that this is an expectation—she as a woman must support the family endeavors, while male family members are credited with the financial success of the family.

Others, with less employment options reported women within the community being limited to working in a chicken processing plant where many AI immigrant women worked and there was very little interaction with the public. This was perceived as a restrictive yet sanctioned method of protecting family or ensuring that the women did not hurt the family honor. While some expressed feeling comfortable to work in an environment among other AI immigrants, they also noted the fact that their activities while at work could be reported back to the community and feared repercussions of unfavorable “talk”.

#### 3.4.3. Domestic Abuse Issues

Women reluctantly discussed issues of domestic abuse, noted to be a taboo subject. Self-blame, victim blaming, and acceptance of abuse as somewhat of a norm were expressed while at the same time suggesting that it was not a very prevalent problem within the community. Minimal if any resources were noted to be available to AI immigrant women in an abusive situation, and many stated that these women would choose to stay for the sake of their children.

The *Gurdwaras* and natal kin were the primary resources abused women were expected to utilize, though fear of “talk” within the family and community was acknowledged as a barrier to accessing these resources. None-the-less, participants expressed the belief that if these resources were successfully sought out (without the knowledge of the abusers), pressure from natal kin or the AI community leaders would be enough to curtail the abusive behavior.

**Table 5:** Qualitative contextual data aligned with variables related to depression.

Qualitative Theme	Quantitative	
**Theme 1: Acculturative Differences by Language**	**Correlates of Themes**
“...because we were raised in India, our society was different. Our in-laws and our parents—we came with those kinds of values. Our kids are raised differently. Their bringing up and our bringing up was different. For them, it will be a burden if I don’t change.” (Punjabi language preference female focus group participant)	Depression was highest among women who completed the survey in Punjabi Anxiety was highly significant for depression among women who completed the survey in Punjabi Among women who completed the survey in English Negative religious coping and anxiety were significant correlates of depression Anxiety was highly significant for depression, although negative religious coping was also a significant variable
“...but my husband was also taking the responsibility back home (India) for whole family…I was raised here (U.S.), even nobody told me, but I knew that’s what’s going to be expected from me…I kept quiet even if I didn’t like it.” (English language preference female focus group participant)	
“In India, I was going to college and I was doing my masters and I was doing research to write a book and I wanted to be a teacher, and I got married and I had to leave my studies. So I wanted more of a career…but then I came here (U.S.) so everything got changed. Since then, I have been in my family, I had my kids, taking care of them and my mother-in-law, she’s a 24-h bed patient and I am taking care of her.” (Punjabi language preference female key informant)	
“So for me there was this fear to even talk to men. I think our own sexuality was affected-like in terms of how do you perceive yourself you know? I’m a woman, and is it OK to even talk to a guy? And be yourself and just feel like a woman? I struggle with that to this day. There is that fear. To me it was fear that was embedded in my conscious.” (English language preference female focus group participant)	
“Yeah definitely as an adolescent it’s depressing, like you know because again you don’t know, you’re growing up in this first generation culture where it’s like your parents don’t understand where you’re coming from. You wanna be more liberal you know, you can’t voice your concerns, you can’t do none of that stuff so it, there will, there can be times that it can be, you know, really depressing and if you don’t have again, somebody to talk to, luckily I had friends and stuff that were going through the same stuff, but as far as family goes, nobody.” (English language preference female key informant)	
**Theme 2: Marriage & Family Conflicts**	**Correlates of Themes**
“…I think a lot of women are expected to be submissive once they get married, because again their parents don’t want that shame.” (English language preference female key informant)	Among women who completed the survey in Punjabi age at marriage, age first pregnancy were significant correlates of depression Among women who completed the survey in English, ideal family size was a significant correlate of depression
“You have to adjust your lifestyle to that of the family you’re marrying into. You have the responsibility of the children, husband. Major change happens.” (Punjabi language preference female key informant)	
“Everything was in the hands of my husband and my mother-in-law, all the decisions, everything, it was between the mother and son…daughter in laws are always the outsiders, okay.” (Punjabi language preference female focus group participant)	
“I came here (U.S.) when I got married and joined my husband’s business….now I have 2 kids so I’m a housewife and a working person, who’s not given any credit because the pay check doesn’t come home.” (Punjabi language preference female focus group participant)	
“…they (in-laws) expect that the daughter-in-law come in and do everything…but it doesn’t work like that, it’s like if there’s more people and just the daughter-in-law is doing the work-that’s a lotttt to do-no one wants to do that.” (Punjabi language preference female key informant)	
**Theme 3: Domestic Abuse Issues**	**Correlates of Themes**
“Abuse, um—I’ve been it, but they don’t say anything. So, if they’re not saying I guess it is OK.” (English language preference female key informant)	Among women who completed the survey in Punjabi domestic violence myth acceptance was a significant correlate of depression
“Before you couldn’t (go outside the family for help), no one wanted that their own family matters and situations be revealed outside. But if there’s a lot of abuse going on, only a brave girl will go out and talk to someone about it. In my cause, I would resolve it at home as much as I can…mostly I would keep it in my own family to resolve. The outside world doesn’t help as much as you think- they tend to just listen and enjoy the entertainment you’re feeding them.” (Punjabi language preference female key informant)	
“That is really messed up, lots of health problems arise when knowing there’s no decent help I can receive. Girls will start to worry knowing that they have wedded into this family, and no one else will be able to take care of us. Even if they hit me or whatever, there’s no one else that can be trusted. If there can be any lady that sees this it could be helpful, but if you come across another man things can get worse…emotionally its very tough.” (Punjabi language preference female key informant)	
“…If there’s a person that they (women in need of help) could talk to and feel safe at the same time doing it, with complete confidentiality and someone who will listen to them- would be something good. That will lessen anxiety, (be) cause lots of problems arise with anxiety…but sadly there is no one like that..“ (Punjabi language preference female key informant)	

Of note, the above themes were either directly or implicitly discussed as situations “others” experienced within their own lives, extended family, or the AI immigrant community resulting in distress. Distress and the associated situations were discussed with some discomfort, as they often involved taboo subjects. Generally, the distress was not discussed as a problem needing a solution, but rather an acknowledgment “of the way things are”.

*“Lots (of women face this problem). Indian women that are already facing lots of problems that do discuss with someone and even they might keep it inside them because they don’t want to create more problems usually end up not saying anything.” (Primarily Punjabi speaking female KI).* 

See Table 5 for additional supporting quotes.

## 4. Discussion

Overall, mental health issues (anxiety and depression) were higher among women than men, and were noted for AI women in both the quantitative and qualitative data. However, significant differences were found when comparing women by language group. Women with English language preference often expressed differences between their own experiences and those they observed among first generation (Punjabi speaking) AI immigrant women. Therefore, we used language preference as a proxy for acculturation in our analyses.

Women with English language preference had on average, mild anxiety and normal scores for depression. Correlates of depression among these women were ideal family size, negative religious coping, and anxiety. Qualitative results support the possibility that ideal family size reflects a discrepancy between generational expectations, causing them acculturative stress. These women expressed a struggle to balance their own desires (to delay childbearing, or choose not to have children, to have an only child or a large family) with the expectations of their families (to start a family soon after marriage, have at least one son, and preferably at least two children).

However, in the multivariate model for these women, only negative religious coping and anxiety remained significant. The contextualization provided by the qualitative results indicate that negative religious coping likely reflects the feeling that the *Gurdwaras* perpetuate the traditional ways of thinking and reinforce gendered role expectations. Some found it difficult to negotiate a successful balance between adopting new ways of being and doing in the U.S. with the expected responsibility of maintaining traditional family roles. They were often caught in the gap between functioning in Western society as model minority AI immigrants (*i.e.*, achieving even as women) and maintaining traditional AI gendered roles. Second generation participants were able to articulate the struggle of this balancing act, which is consistent with women feeling torn between the child-rearing practices of Indian and the U.S. [44]. AI immigrant women striving to be “good Asian Moms” must renegotiate their own motherhood around more Western parenting constructs. Indeed, other studies have noted that AI immigrant women often bear the responsibility of ensuring cultural continuity and family stability, despite this conversely hampering their personal freedom [14]. Our participants indicated that fear of “talk” prohibited their freedom of self-expression, restricted adoption of less gendered roles, and increased distress. Thus, while the overall mean depression scores for this group of women was within normal limits, when they do experience depression, it is primarily expressed as anxiety.

Anxiety was higher among women with Punjabi language preference, and they also presented with a significantly higher mean for depression (in the mild to moderate range). Correlates of depression among these women were early age at marriage, early age at first pregnancy, acceptance of domestic violence myths, and anxiety. In the multivariate model, however, the only significantly related variables that remained were early age at first pregnancy and anxiety. These results were validated in the qualitative data which indicated that early childbearing further isolates Punjabi speaking women to traditionally gendered roles (such as their responsibilities in the home), and limits the prospect of opportunities to pursue personal education, employment, or professional goals, as well as limiting social interactions outside of the home or AI community. Indeed, these women usually having been separated by marriage from their natal kin who remained in India, often perceived a lack of social support, which they expressed as isolation and loneliness. This finding is consistent with other literature indicating that new mothers who are first generation immigrants often perceive motherhood to be a source of stress because of severed ties to “home” resulting in less or different social support than they had expected [44].

The Punjabi language preference women were also more often married at a younger age and brought to the U.S. as wives of AI men who were either second generation or had immigrated some years earlier. They often had to leave their education unfinished or had completed a degree—leaving behind their family and friends, and the lives they knew up until that point. Once they arrived in the U.S. many found themselves isolated either within their homes or the AI community due to limited understanding or ability to speak English, lack of familiarity with U.S. society, inability to drive, and the expectation that they would devote themselves to husband and home. Devotion to the home in a joint family (living with husband and his parents) included subservience to in-laws, being “overseen” by the mother-in laws, conceiving soon after marriage, doing household chores and maintaining tradition.

Some were also expected to work outside the home to contribute to the family income, but were limited in where they could work by a number of factors. Limited English precluded some from pursuing the higher paying or professional jobs they were otherwise qualified for. Some women were only allowed to work for extended family members. Still others found that sociocultural expectations determined that they could not work in an environment that allowed them to interact with the public; therefore, they were restricted to working in enclosed environments primarily with other AI immigrant women, such as a chicken processing plant. Balancing work and family is a burden to AI immigrant women. In India, traditionally gendered distribution of work and family roles is slowly changing [45], however, traditional expectations continue to influence AI immigrants experiences. While for some there may be a feeling of empowerment related to working outside of the home, the commitment to family roles is most strongly emphasized in Indian culture as being of utmost important and a requirement of social expectation [46].

Despite the differences noted between these subgroups (based on language preference) there were also shared experiences. All women, regardless of language and first or second generation status, are subject to meeting the expectations of husbands and in-laws, and both subgroups expressed the constant fear of “talk” within the community reflecting the elevated anxiety in both groups, although it was even higher among Punjabi speaking women. Negative “talk” was feared if they were perceived as having failed to meet these expectations, which would result in shame or loss of family honor.

Additionally, across both subgroups, regardless of language preference, domestic abuse was a taboo subject and was discussed as something that happened to others, as having happened to someone they only heard about or knew of from a distance, even when the investigators felt it was very likely spoken of from personal experience. Reported reasons for abuse included a confluence of factors, described by most as situations when sociocultural, economic, interpersonal, and family expectations were not met. Yet the occurrence of domestic violence was acknowledged with a sense of resignation. For those who admitted to personally having known someone or themselves having experienced abuse, the sense of fatalism was pronounced. Rarely was the abuse considered severe enough to warrant action by others, and ways of dealing with the situation within the community were acknowledged as very ineffective.

Despite the challenges noted above, our participants showed many strengths. Resiliency was noted in their determination to thrive in the U.S., devotion to family (especially children), and adaptation to U.S. culture while maintaining religious beliefs and AI community connections, as well as passing on cultural heritage to their children. Even though family issues and the sociocultural expectations of the AI community were stressors, they were also often seen as a source of support, strength, acceptance, and protection. In line with other studies, AI women may be more inclined to utilize informal support mechanisms such as these, rather seeking mental health care [7,47]. Acculturation balanced with maintaining AI traditions was expressed as evidence of resilience and ultimate achievement, which results in wellbeing and satisfaction with life.

Limitations to be noted include the cross-sectional nature of the study, possible self-report bias, and generalizability. However, we tried to counter-balance this limitation using qualitative data to contextualize the responses. This study was done in a specific AI population in Central California and there may be differences in other AI immigrant populations living elsewhere in the U.S. Strengths of the study include that both the qualitative and quantitative data were collected in English and Punjabi according to participant preference, and the used of mixed-methods.

Although we only found high-normative to low levels of depression in the quantitative data, depression was omnipresent in the qualitative data collected from key informant and focus group participants—while never overtly named, distressing situations and mental health symptoms such as anxiety and depression were described. We believe this reflects the sociocultural stigma attached to mental health issues in the AI community, as found in other studies [48,49]. The quantitative data, which was otherwise pristine from a data completeness standpoint, had missing data only in areas where we asked about depression, anxiety, and domestic violence. Qualitative data collected from community stakeholders, including Gurdwara leaders and health care providers, confirmed that depression was a widespread problem but never talked about.

“...It takes a long time for a Punjabi woman to see a psychiatrist, to get help. As I said it’s a taboo…” (KI-Punjabi health care provider).

Thus, despite having gained entry to the community with a gender, language, and culturally matched investigator as a member of our research team whose family is well-known and respected within the community, community involvement and the support of community leaders, it was difficult to obtain mental health data. Indeed, the topic of mental health is so highly stigmatized that even on an anonymous survey, rather than giving a false response (which would likely have created a favorable response bias), our participants could not bring themselves to complete these sections. Without the use of mixed methods, our understanding of depression among AI immigrant women in the community would have been far more limited.

Mixed methods allowed a more comprehensive understanding of depression among our sample of AI immigrant women. Quantitative results enabled us to identify the subgroup with highest levels of depression (women who chose to complete the survey in Punjabi), and distinguish different correlates and predictors of depression by subgroup (English or Punjabi preference). Qualitative results highlighted nuances enabling us to understand the difference in factors experienced by these two subgroups, as well as giving an indication of the severity of mental health stigmatization and the prevalence of the depression.

## 5. Conclusions

Research that utilizes mixed methods is essential to uncovering hidden mental health disparities in model minority populations such as AI immigrant groups. Cultural stigmatization of mental health issues, such as depression, and the desire to live up to the model minority stereotype inhibit responses to screening tools and quantitative surveys. Therefore, qualitative data collected in a culturally sensitive fashion begins to help determine the magnitude of mental health issues. We strongly encourage others doing mental health research among immigrant populations, especially model minorities (such as AI) consider the challenge of underreporting, and utilize mixed methods to contextualize their results.
